# Male-like sexual behavior of female mouse lacking fucose mutarotase

**DOI:** 10.1186/1471-2156-11-62

**Published:** 2010-07-07

**Authors:** Dongkyu Park, Dongwook Choi, Junghoon Lee, Dae-sik Lim, Chankyu Park

**Affiliations:** 1 Department of Biological Sciences, Korea Advanced Institute of Science and Technology, 335 Gwahangno, Yuseong-Gu, Daejon 305-701, Korea

## Abstract

**Background:**

Mutarotases are recently characterized family of enzymes that are involved in the anomeric conversions of monosaccharides. The mammalian fucose mutarotase (FucM) was reported in cultured cells to facilitate fucose utilization and incorporation into protein by glycosylation. However, the role of this enzyme in animal has not been elucidated.

**Results:**

We generated a mutant mouse specifically lacking the fucose mutarotase (FucM) gene. The *FucM *knockout mice displayed an abnormal sexual receptivity with a drastic reduction in lordosis score, although the animals were fertile due to a rare and forced intromission by a typical male. We examined the anteroventral periventricular nucleus (AVPv) of the preoptic region in brain and found that the mutant females showed a reduction in tyrosine hydoxylase positive neurons compared to that of a normal female. Furthermore, the mutant females exhibited a masculine behavior, such as mounting to a normal female partner as well as showing a preference to female urine. We found a reduction of fucosylated serum alpha-fetoprotein (AFP) in a mutant embryo relative to that of a wild-type embryo.

**Conclusions:**

The observation that *FucM*^-/- ^female mouse exhibits a phenotypic similarity to a wild-type male in terms of its sexual behavior appears to be due to the neurodevelopmental changes in preoptic area of mutant brain resembling a wild-type male. Since the previous studies indicate that AFP plays a role in titrating estradiol that are required to consolidate sexual preference of female mice, we speculate that the reduced level of AFP in *FucM*^-/- ^mouse, presumably resulting from the reduced fucosylation, is responsible for the male-like sexual behavior observed in the FucM knock-out mouse.

## Background

Fucose mutarotase (FucM) is an enzyme that is involved in the anomeric conversion of L-fucose [1] in the salvage pathway for GDP-fucose synthesis, which facilitates the incorporation of L-fucose into protein. The importance of fucosylation has been exemplified by the case of human congenital disorders of glycosylation (CDGs). CDGs, classified as CDG types I and II, involve an impaired glycosylation with complex pathologies of developmental alteration in the brain, liver, and immune system. CDG-IIc results from a defect in GDP-L-fucose transport into Golgi [2-4]. GDP-L-fucose is formed either by the de novo or salvage pathways, which in Golgi serves as a substrate for the glycosylation mediated by different fucosyltransferases. Mice deficient in FX, an enzyme synthesizing GDP-fucose from GDP-mannose as part of the de novo pathway, exhibit embryonic lethality, premature death, or infertility due to the deficiency of cellular fucosylation [5]. Interestingly, this phenotype can be rescued by a fucose supplement.

The sexual differences in mice are characterized by their reproductive behaviors, e.g. mounting or receptivity. Feminization in sexual dimorphism is believed to be a default state that leads to a neural substrate conducive to female reproductive behavior [6]. The loss of female-like characteristics can be assessed by examining tyrosine hydroxylase expression in the anteroventricular nucleus of the preoptic region (AVPv) [7-9] as a sexually dimorphic marker. According to the classical view, sexual differentiation in the brain occurs under the influence of testosterone and/or estradiol derived from neural aromatization of testosterone: the brain develops as male in the presence of these steroid hormones, and as female in their absence. Consistently, recent studies suggest that the estrogen produced by the placenta during late gestation can affect sexual differentiation into female unless it is not titrated by alpha-fetoprotein (AFP) that has an affinity to estrogen [10-13]. The role of AFP in the development of the female brain has been demonstrated by an observation in which knockout mice lacking alpha-fetoprotein were infertile [14] due to an impairment of the hypothalamic/pituitary system and to a failure in the estrous cycle [14,15]. The consequence was a complete absence of ovulation in the homozygous *Afp*-knockout mouse [14] and a behavioral change among the females that was indicative of defeminization [9]. AFPs contain carbohydrate moieties and thus exist in heterogeneous isoforms, whose synthesis decreases dramatically after birth with trace amounts of expression in the adult liver. Human AFP contains a single N-linked glycan, whereas murine AFP has two N- and one O-glycans [16]. Estradiol binding was only reported for murine AFP [17-19].

In this study, we generated mutant mice specifically lacking the *FucM *gene that encodes fucose mutarotase. The homo- and heterozygous mutant females exhibited deficits in sexual receptivity as demonstrated by a drop in lordosis score. Moreover, the mutants displayed masculine behaviors and preferences to female urine over male urine. The possible association of such behavioral change with an altered level of alpha-fetoproteins in *FucM*^-/- ^mice is discussed.

## Results

### FucM-/- results in a change in female sexual receptivity

**Figure 1:**
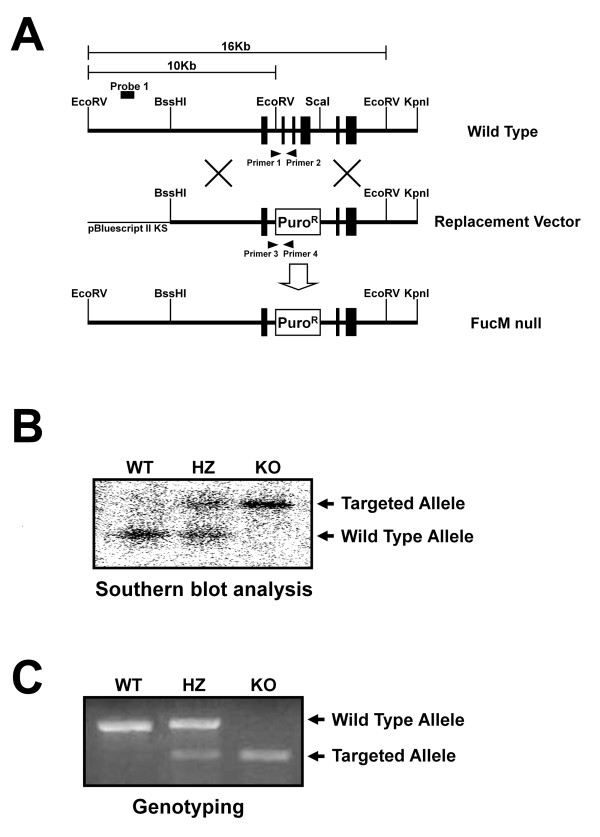
**Targeted Disruption of the *FucM *Gene**. (A) Schematic diagram for the targeted disruption of the *FucM *gene. The structures of the targeting vector, wild-type, and disrupted *FucM *alleles are shown. The solid boxes represent the exons (a total of six in the wild-type). The restriction sites used for constructing the targeting vector and for analysis by Southern blot are EcoRV, BssHI, ScaI, and KpnI. A puromycin expressing cassette was inserted between the EcoRV and ScaI sites, eliminating exons 2-4 and the EcoRV site of the WT gene. (B) Southern analysis for genomic DNA from the wild-type (+/+), heterozygous (+/-) and *FucM *null (-/-) mice. The expected sizes of the wild-type and disrupted *FucM *alleles are 10 and 16 Kb, respectively, which were detected by Southern blotting with EcoRV digestion and hybridization using the probe 1 shown in (A). (C) The wild-type (primer 1 and 2) and disrupted alleles (primer 3 and 4) were also detected by PCR.

We generated *FucM*^-/- ^(KO) mice by replacing exons 2, 3, and 4 of the fucose mutarotase gene (*FucM*) with a puromycin resistance gene using embryonic stem (ES) cells derived from a 129x1/SvJ mouse (Figure 1A). The correct gene targeting was confirmed by Southern blotting (Figure 1B) and also by PCR (Figure 1C) with the primer sets used to detect the wild-type (primer 1 and 2) and knockout alleles (primer 3 and 4). *FucM*-targeted clones were injected into C57BL/6 recipient blastocysts, and the obtained chimeras were crossed with C57BL/6 partners. The heterozygous mice for the *FucM *allele (*FucM*^+/-^, HZ) were crossed to generate homozygous *FucM*^-/- ^mice. The hetero- and homozygous knockout animals did not show any indication of embryonic lethality with statistically significant Mendelian segregation ratios. The most apparent abnormality was a mutant female avoiding a normal male partner in the regular mating cage, while a slight loss in body weight and minor behavioral change were occasionally observed for the hetero- or homozygous mutant animals (data not shown). Thus, we systematically analyzed the reproductive behaviors of *FucM *mutant mice.

### Mutant female suppresses lordosis, with occasional mating by forced intromission

**Figure 2:**
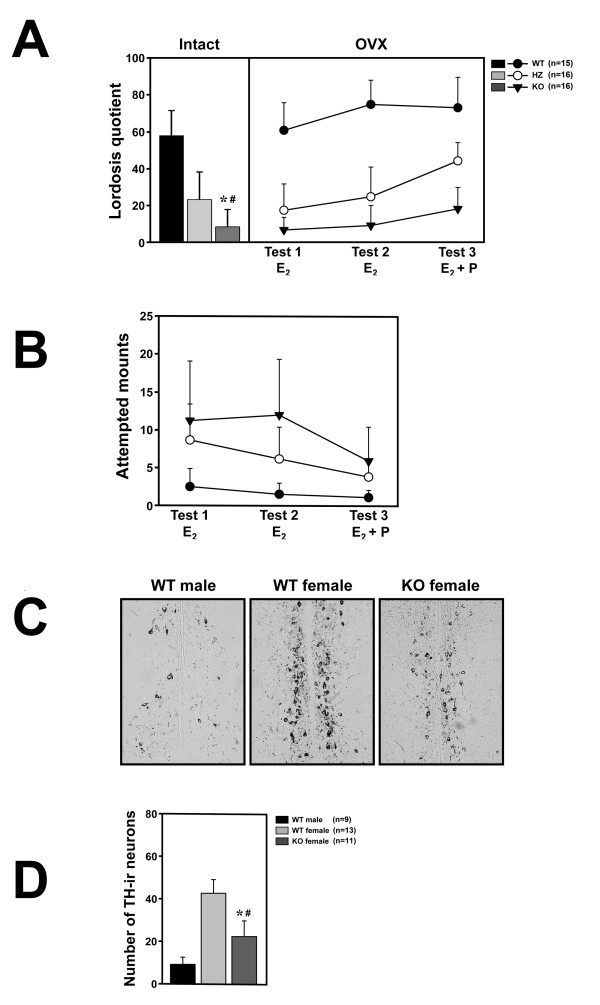
**Sexual behaviors of wild-type and *FucM *knockout females**. (A) Lordosis quotients of gonads-intact (left) and ovariectomized females (right). Lordotic responses by three consecutive tests were observed for the ovariectomized wild-type controls and *FucM *gene-disrupted mice after treatments with E_2 _(test 1 and 2) and E_2 _plus progesterone (test 3). The tests were carried out at a one-week interval. The scores of the KO females were different with *P *< 0.001 (*) from that of the wild-type and *P *> 0.05 (#) from that of heterozygote. (B) Frequencies of attempted mounts, mounts and intromission-like behaviors in three consecutive tests of subject females with a female partner. (C) Photomicrographs of sections in AVPv stained by immunohistochemistry for tyrosine hydroxylase in males, wild-type females, and *FucM *KO females. (D) Numbers of tyrosine hydroxylase-immunoreactive (ir) neurons in the sections shown in C: *, *P *< 0.001 compared to that of the wild-type males; #, *P *< 0.001 compared to that of wild-type females. Data are shown as mean ± s.e.m.

For testing the female-typical sexual behavior of *FucM *mutants, we observed both gonad-intact and ovariectomized (OVX) females. The gonad intact females were assessed for their stages of estrous cycle using vaginal smears in order to select individuals (*n *= 13) at the estrus stage. The wild types (WTs) and *FucM *mutants did not exhibit any difference in hormonal cycle. The ovariectomized females (WT, *n *= 15; HZ and KO, *n *= 16) were subcutaneously implanted with an osmotic mini-pump in order to infuse them with 17β-estradiol (E_2_) in sesame oil, and were tested three times on a week interval. For the third trial, the mice were injected with 500 μg of progesterone four hours before the tests. Female sexual receptivity was measured by calculating the lordosis quotient (multiplied by 100) as the number of times a female exhibited lordosis divided by the number of mounts. There were no genotype differences in the total number of social contact behaviors such as sniffing, chasing, or touching of the bodies of the females by a wild-type stud male. However, in the sexual behaviors of the stud males, such as attempted mounts, mounts, and intromissions, clear differences were found between genotypes, particularly because of rejection by females. About 59, 11, and 5% of the WT, HZ, and KO gonad-intact females, respectively, showed sexual receptivity at least once during a 20 min test period (Figure 2A).

One-way ANOVA with genotype as the independent factor indicates the significance of differences between genotypes [*F*(2,36) = 137.68, *P *< 0.001]. The receptivity of the KO females was comparable with that of HZs (P > 0.05), but significantly lower than that of the WTs (P < 0.001) by a *post-hoc *Fisher probable least-squares difference (PLSD) comparison. The change in sexual receptivity, i.e. reduced lordosis, observed in the *FucM *mutants was further verified for ovariectomized females with a treatment of sexual hormone (E_2_). The ovariectomized HZ and KO females displayed sexual receptivity lower than that of the WTs in response to a male suitor (Figure 2A). Repeated measures ANOVA indicate that there is a significant effect of genotype on lordosis quotients [*F*(2,44) = 166.57, *P *< 0.001] and on repeated testing [*F*(2,88) = 25.87, *P *< 0.001], as well as on the interaction between these factors [*F*(4,88) = 4.41, *P *< 0.001]. Although the mean lordosis quotients in ovariectomized KO females were slowly increased with time, they were significantly lower than that of the wild-type females in all trials. Interestingly, the HZ females displayed an intermediate lordosis response, which was significantly different from that of the WTs and KOs by the *post-hoc *comparisons (*P *< 0.001 compared to the WTs or KOs), likely reflecting a dose-dependent effect of fucose mutarotase on behavior.

### Male-typical coital behavior displayed by the mutant mice

The lack of female receptivity in *FucM *mutants further led us to test whether the KO females exert male-typical sexual behavior, which was examined over three weeks by placing an E_2_-stimulated female partner of WT into the home cage of an ovariectomized female (WT, *n *= 13; HZ, *n *= 12; KO, *n *= 10). The mutant female mice showed masculine behaviors, such as attempted mounting, mounting, and pseudo-intromission, towards an estradiol-primed partner female (Figure 2B). The male-typical behaviors were scored for 30 min. For the third trial, the ovariectomized females were injected with 500 μg of progesterone. Repeated measures ANOVA indicated that the frequencies of attempted mounts and actual mounts of female partners by tested females were significantly higher than that of the control females [*F*(2,32) = 15.03, *P *< 0.001], and there was a significant effect of repeated testing [*F*(2,64) = 16.49, *P *< 0.001] as well as a significant factor interaction [*F*(4,64) = 6.15, *P *< 0.001]. Differences between groups were obtained by Fisher's *post hoc *analysis, in which the KO females can be compared to the WT and HZ females (*P *< 0.001) with significance. Unlike the lordosis behavior, the male-typical coital behavior of the mutant females were notably reduced by the hormone (E_2_) treatment.

### Reduced tyrosine hydroxylase expression in the *FucM *mutant

The populations of tyrosine hydroxylase (TH)-expressing hypothalamic cells are sexually dimorphic in rats and mice [20,21]. TH-immunoreactive (ir) positive neurons in the anteroventral periventricular nucleus (AVPv) of the preoptic region regulating gonadotropin release are necessary for female ovulation, whose numbers in a female, in addition to their size difference, are two to four times greater than in a male [7,20-22]. Ovariectomy of the female does not affect TH expression, while chronic treatment with high levels of estradiol reduces its expression [7,20]. When we examined the tyrosine hydroxylase positive neurons in the AVPv in the control and mutant mice by immunohistochemistry, the *FucM *KO female mice exhibited less TH-immunoreactive cells in the AVPv than the normal female mice, albeit slightly more than those of the normal males (Figure 2C and 2D). One-way ANOVA indicates significant differences between the groups [F(2,30) = 18.57, P < 0.001]. The apparent lack of knock out phenotype in male mouse seems to be consistent with an indistinguishable pattern of TH-ir neurons in the mutant male from that of wild-type male in the AVPv region of the brain (data not shown).

### *FucM *mutant female prefers female urine

**Figure 3:**
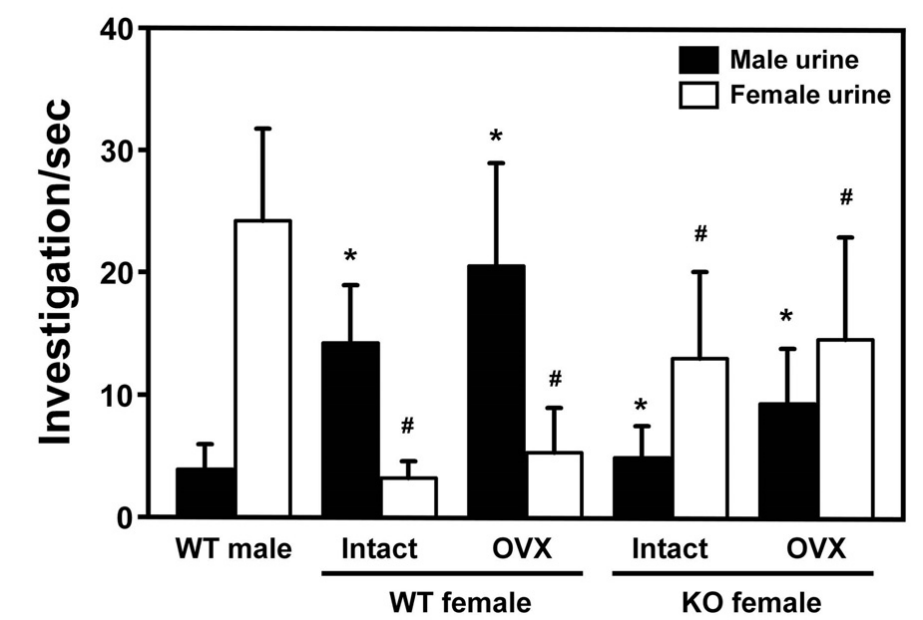
**The olfactory response of female mice lacking *FucM***. The mean time spent sniffing male and female urine by wild-type males (*n *= 15), wild-type females (intact, *n *= 15; OVX, *n *= 13) and *FucM *KO females (intact, *n *= 12; OVX, *n *= 10). Result of *post-hoc *comparisons by the Fisher PLSD test are indicated as follows: *, P < 0.001 when compared to wild-type males for male urine; #, P < 0.001 when compared to wild-type males for female urine. Data are expressed as mean ± s.e.m.

The key to gender-specific behavior in mice is a cluster of receptors in their noses, which allows them to smell pheromones, eliciting a sexual signal among members of the same species. Male and female mice are motivated to approach the opposite sex by specific responses to urinary odors [23]. We investigated the olfactory responsiveness of the *FucM *mutant to the urinary odors of estrous females. The urine preference tests were carried out with simultaneous presentation of male and female urine. The time that the subject mice spent sniffing male or female urine through nasal contact was scored for 5 min. Preference of the WT females for male urine were noted, while the WT males and KO females preferred female urine (Figure 3). ANOVA of the preference for female urine indicated a significant effect of genotype [*F*(2,39) = 47.24, *P *< 0.001], with significant results of Fisher's *post-hoc *analysis for the KO females compared to the WT males and females (*P *< 0.001). Also, ANOVA of the preference for male urine indicated a significant effect of genotype [*F*(2,39) = 42.15, *P *< 0.001]. Destruction of olfactory epithelium by ZnSO_4 _eliminated any sniffing of stimulating urine (data not shown).

### Deficiency in fucose mutarotase reduces serum level of fucosylated alpha-fetoprotein

From the male-like behavioral change of *FucM *mutant females and the corresponding alterations in the neural substrate, one can imagine that a change in sexual differentiation is responsible for the phenotypic difference. One likely candidate eliciting such change is alpha-fetoprotein, recently implicated in mediating a hormonal effect on brain development [9,15] associated with sexual behavior, during the late gestation to birth. Since fucose mutarotase affects the intracellular level of GDP-fucose, and thus fucosylation, an immediate consequence of a *FucM *defect would be a change, i.e. reduction, in AFP fucosylation. We investigated the fucosylation status of whole protein and serum protein, focusing on AFP, during embryogenesis using two-dimensional electrophoresis and a MALDI-MS analysis of proteins from an embryo at 16.5d postcoitum (dpc), indicating that AFPs are a major group of proteins being expressed during gestation in both WT and KO mice.

**Figure 4:**
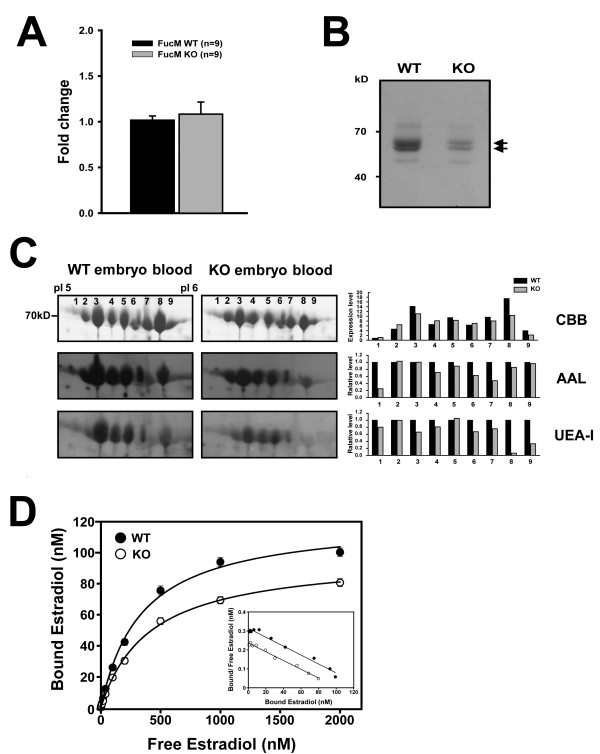
**Amounts of AFP and its binding to 17β-estradiol in embryonic sera**. (A) Gene expression analysis of AFP in WT and KO embryos at E16.5. The amplified copies of AFP were normalized against GAPDH, from which the ratio between copy numbers was determined to obtain normalized fold change. (B) Fetal mouse sera containing AFP (arrow) were visualized by 10% SDS-PAGE, followed by Coomassie brilliant blue (CBB) staining, which were obtained from 25 each of wild-type and mutant embryos at E16.5. They were diluted 1/30 and loaded. (C) Embryonic serum proteins from the WT and the *FucM *deficient mice were subjected to 2-D electrophoresis, followed by CBB staining. After blotting onto a nitrocellulose membrane, lectin blotting was performed. *Aleuria aurantia *lectin (AAL) binds to *N*-linked oligosaccharides with L-fucose linked (α-1,3/α-1,6) to the proximal GlcNAc residue, while *Ulex europaeus *agglutinin I preferentially binds to Fuc*α*(1-2)Gal*β*(1-4)GlcNAc. Spot intensities were analyzed using ImageJ to estimate relative fucosylation levels of AFP. (D) Scatchard analyses for the interactions of 17β-estradiol (E_2_) with mouse embryonic sera are shown, which were determined by the dextran-coated charcoal (DCC) technique as described in the Methods section.

When we monitored gene expression levels of AFP by real-time PCR in KO and WT, there was no significant difference between WT (*n *= 3) and KO (*n *= 3) embryos (Figure 4A). However, the absolute levels of AFPs are lower in a KO embryo (Figure 4B), presumably reflecting an inefficient secretion into the serum [24] or a problem with turnover. We further analyzed amounts and fucosylations of the AFP isoforms by separating on 2D gel electrophoresis (Figure 4C). The total amounts of Coomassie-stained proteins are higher in WT than that of KO, which is consistent with the data in Figure 4B. When we assessed fucosylations in different AFP isoforms by blotting with the AAL and UEA-I lections, recognizing α-1,6/α-1,3 and α-1,2 linkages, respectively, the signals are generally lower in the KO embryo compared to that of wild type, although there are variations in different isoforms. Since the fucosylation signals were standardized by the amounts of Coummassie-stained proteins, this reduction indeed indicates overall decrease in AFP fucosylation.

### Embryonic levels, not affinity, of AFPs are responsible for estradiol titration

In order to test whether the different amounts of fucosylated AFP correlate with estradiol bindings, a serum concentration of AFP was calculated by 2D-western blotting, and the affinity constants for E_2 _of the serum was investigated using saturation analysis in 16.5 dpc embryos (Figure 4D). The equilibrium association constant (K_a_) and maximal binding capacity (B_max_) were estimated by Scatchard plotting. Blood from each embryo was taken by heart puncture, from which the same volumes of sera after removing the blood cells were used to determine the binding activities for E_2 _and for western analysis. In fetal mouse serum, AFP and albumin are the major proteins, among which only AFP exerts an E_2 _binding activity [18]. Because the binding activity of AFP for E_2 _depends on conditions [18,25] such as carbohydrate modifications, embryonic stage (15-18 days of gestation) and purification methods, crude serum samples were used for monitoring the *in vivo *states affecting brain development.

As shown in Figure 4D, the K_a _values for E_2 _in crude embryonic sera were similar (WT; ~0.24 × 10^7 ^M^-1 ^and KO; ~0.23 × 10^7 ^M^-1^), although the B_max _for E_2 _in the controls differs from that of the mutants. The differences in B_max _for E_2_, but not in K_a_, appear to reflect the differences in the amount of AFPs (Figure 4B, 4C) present in the sera.

## Discussion

We investigated the role of fucose mutarotase by generating mice in which the *FucM *gene was selectively ablated. The mutant mice were healthy without any indication of anatomical abnormalities. The most distinct phenotype observed with the mutant female mice was a significant reduction in hormone-stimulated sexual receptivity measured as a lordosis quotient. Although the homozygous mutant females exhibited altered sexual behaviors, i.e. reduced lordosis and male-typical coital behavior, they were fertile with typical estrous cycles and ovulation, similar to that of a normal female. Most of the mutant females became pregnant, and an average of 8.6 pups was produced per litter. The successful mating of the KO females was probably due to the forced mating attempts of sexually experienced males despite rejections by the females. We did not observe differences in maternal behavior between WT and KO females (data not shown). Proceptivity behaviors such as solicitation were also avoided by the KO females (data not shown). In the case of AFP knockout mouse, the female exhibits a defect in ovulation, in addition to a change in sexual preference of female [9]. A sole alteration of behavioral phenotype in *FucM *knockout mutant, while preserving its fertility, might lie in different degrees of estrogen titration by AFP, i.e. lack of AFP versus the reduced level of fucosylated AFP, during embryonic development for sexual dimorphism. Although the knockouts of estrogen receptors, ERα and ERβ, show either one or both of the phenotypes, abnormal sexual receptivity and fertility [26,27], the mechanism underlying these alterations might be different due to its postnatal nature of manifestation. The behavioral abnormality in female knock-out mouse was observed in the progenies with the mixed genetic background made between the 129sv and C57BL/6 inbred lines as usually did in most knock out studies. Thus, it would be desirable in later investigation to observe a phenotypic persistence in homogeneous genetic background after a series of inbreeding as demonstrated previously in the experiment with AFP knock-out mouse [9].

Since a *FucM*^-/- ^female mouse exhibits a phenotypic similarity to a wild-type male in terms of its sexual behavior, we speculated that the reproductive circuitry of the mutants might have altered to resemble that of a wild-type male mouse. Since the circuitry starts from sensing a pheromonal cue in olfactory system [28,29], we carried out a urine preference test to investigate the abnormality in the mutants. The time spent by KO females sniffing female urine was comparable to that of the WT males, which was significantly longer than that of male urine (Figure 3). Since the olfactory partner preference is sexually dimorphic and organized by perinatal exposure to gonadal steroids [30], it is possible that the brain of *FucM*^-/- ^females might have experienced a male-like sexual differentiation during development. The sexual differentiation of the female brain occurs during a critical period of development, in which sexually dimorphic neural populations are organized by testosterone or its metabolite estradiol [6]. The AVPv of the hypothalamus is sexually dimorphic and the neurons that express tyrosine hydroxylase (TH) are more abundant in that of the female [31]. When we analyzed the sexually dimorphic populations of tyrosine hydroxylase (TH)-immunoreactive neurons in the AVPv nuclei of *FucM*^-/- ^and wild-type littermates, an alteration in the forebrain system of the *FucM*^-/- ^females was observed, i.e. a lower number of TH-positive neurons as in the wild-type males.

Feminization is known to be induced by blocking estrogen action during embryonic development, which is achieved by an alpha-fetoprotein binding to circulating estradiol in the female fetus [9,32]. However, defeminization can occur by an increased level of estradiol [6,33,34], and perhaps masculinization by affecting cyclooxygenase-2 (COX-2) regulation to increase prostaglandin E_2 _(PGE_2_) [35]. In our study, an altered level of fucosylated alpha-fetoproteins was detected in the *FucM*^-/- ^mice, which was analyzed by 2D gel electrophoresis and lectin blotting for fucosylated proteins, followed by MALDI-TOF MASS fingerprinting (Figure 4C). Thus, it is tempting to speculate that the altered level of serum alpha-fetoprotein might be responsible for sexual abnormality in the *FucM *KO mice. Since the level of fucose mutarotase is reported to affect the intracellular level of GDP-fucose, and thus fucosylated proteins [1], the altered level of AFP is very likely to result from different fucosylations.

The AFP isofoms we detected are similar to that reported previously in hepatocellular carcinoma (HCC) and nonseminomatous germ cell tumors (NSGCTs) patients [36]. We observed that fucosylations of AFP isoforms were lower in *FucM *KO mouse than that of WT mouse, although some AFP isoforms were essentially unchanged (Figure 4C). Since protein containing glycans with more sialic acids (SAs) have lower pI and higher molecular mass, the AFP spots are displayed as a descending row on two-demensional gel. Differences in lectin signal by AAL and UEA-I in WT and KO AFP isoforms might be explained by altered fucosylation efficiency due to differences in their glycan structures. Although there is no direct evidence on the effect of fucosylation on either secretion or stability of AFP, it is likely to happen *in vivo *based on previous results where the folding and stability of proteins were modulated by other types of glycosylations [24,37,38]. For example, fucosylation and further elongation of saccharide are critical for the activity of Notch [39,40]. The fucosylation of glycoproteins, including α1-antitrypsin (AAT), hepatoglobin (Hp), and α1-acid glycoprotein (AGP), may facilitate their secretion into bile ducts in the liver through an apical surface of human hepatocytes [41]. Also, fucosylation of synapsin critically affects its expression and turnover in presynaptic nerve terminals [42].

The maximal level of AFP in a human fetus is about 3 mg/ml, which decreases to a basal level of approximately 2-20 ng/ml in an adult [43]. However, this level went up to 342 ng/ml in patients with hepatocellular carcinoma [44]. In adult mice, the basal level of AFP is somewhat higher than in human sera, and is variable in different inbred lines. BALB/c/J mice have 994 ng/ml concentrations of AFP on average, while other strains have between 34 and 173 ng/ml [45]. In our estimation, the AFP concentration of a wild-type embryo was about 5.1 mg/ml, whereas it decreased to about 23.5% in a *FucM*^-/- ^KO embryo. Since the total amount of serum estradiol in 18d embryonic mice is about 90 pg/ml with free estradiol of less than 1 pg/ml [46], presumably being regulated by the amount of AFP [32], the observed difference in the amounts of AFP in embryonic sera might be critical in titrating estradiol associated with defeminization. In our study, the level of AFP in serum was decreased in *FucM*-deficient embryo, although change in the levels of hepatic AFP was not observed (data not shown), reflecting the difference in B_max _for E_2_, not on the binding affinity (K_a_). This may imply that the fucosylation of AFP may not be a signal for its secretion into the serum. Alternatively, the carbohydrate portion of AFP might be involved in modulating the serum concentration of AFP as in the case of the desialylated mouse AFP that binds to the asialoprotein receptors, which is subject to be endocytosed and degraded [47].

## Conclusions

The study demonstrates that *FucM*^-/- ^female mouse exhibits a phenotypic similarity to a wild-type male in terms of its sexual behavior. We speculate that this behavioural change is likely to be related to the neurodevelopmental change in preoptic area of mutant brain that became similar to that of wild-type male. Since the level of AFP, playing a role in titrating estradiol, is known to affect development of female brain associated with sexual preference, we speculate that the lower level of AFP, presumably resulting from the reduced fucosylation, in the mutant embryo causes abnormal brain development. Our studies on the *FucM*^-/- ^mouse may provide an animal model for understanding sexual preference of animal at genetic as well as epigenetic levels.

## Methods

### Targeted deletion of FucM gene and the generation of mutant mice

The murine *FucM *gene (LOC69064) was isolated from a 129x1/SvJ genomic BAC (bacterial artificial chromosome) library (Research Genetics). A PGK-puro expression cassette, including the *puro *gene being transcribed by the mouse phosphoglycerate kinase (PGK) promoter with the PGK poly(A) addition site, was obtained from the pBluescript II KS (+). An approximately 5.1 kb BssHI/EcoRV genomic fragment upstream of the exon 2, and an approximately 4.8 kb ScaI/KpnI genomic fragment of the *FucM *gene including the exons 5 and 6, as shown in Figure 1A, were filled in by Klenow polymerase. To generate a targeting construct, the genomic fragments were subcloned into the *puro *expression cassette of the PGK-puro target vector. A *diphtheria toxin A *(DTA) cassette for negative selection was flanked to the 3' end of the *FucM *sequences by cloning into the NotI site. The vector was linearized with SalI digestion prior to electroporation. E14K embryonic stem (ES) cells, derived from 129x1/SvJ mice, were cultured on mouse embryo fibroblast feeder cells that are mitotically inactive and puromycin-resistant. The cells were electroporated with a linearized targeting vector and screened for homologous recombination by Southern blot hybridization using the 5' flanking probe 1 shown in Figure 1A. Two targeted clones were detected out of the 340 clones screened. A targeted ES clone was injected into C57BL/6 blastocysts to generate chimeras. The chimeras were tested for germline transmission of the ES cell by mating with C57BL/6 mice; offspring with an agouti coat color must have been derived from the targeted ES cells, indicating germline transmission. The heterozygous mice were interbred to obtain mice homozygous for the disrupted *FucM *gene.

### Animals

The *FucM *KO mice and their WT and HZ littermates from a mixed background of C57BL/6 and 129x1/SvJ were used. The mice were housed in a specific pathogen-free (SPF) facility operating in a controlled air-filtered environment with autoclaved water, cages, and bedding, and were fed sterilized Purina rodent chow. All of the mice were raised in Space Saver' cages (Model 1145T, Tecniplast, Buguggiate, Italy) in temperature-controlled facilities at 22°C under 12 h of a light-dark cycle with the light on at 07:00. Humidity was maintained at 55% with food and water freely available. All manipulations of the mice were performed in accordance with NIH guide lines for the Care and Use of Laboratory Animals.

### Test for sexual receptivity

All male and female mice were housed with parents before weaning (25 days). The female subjects were housed together prior to ovariectomy. The ovariectomized female mice were housed individually prior to behavior experiments. The female mice at the ages of 10-12 weeks were used to test feminine sexual behavior and male coital behavior. All experiments began 1 hr after the start of dark cycle (7 pm) in a Space Saver' cages (Width 30 cm × Depth 13 cm × Height 20 cm) in SPF facility. As a preliminary step for sexual receptivity tests, the stud males, chosen by screening sexually vigorous male mice, were placed in the presence of a 17β-estradiol (Calbiochem) primed female in an adjacent chamber, in order for an exposure to visual, auditory, and olfactory cues without any physical contact [48]. Males were allowed to habituate in their new environment for at least 15 min prior to the start of the test. Gonad-intact females were selected at the estrus stage using vaginal smear and Papanicolaou (PAP) staining [49]. In adulthood, stimulus females were ovariectomized using Avertin Anesthesia and received an Alzet mini-osmotic pump (model 2004, Durect Corp., Cupertino, CA, USA) containing 1.6 μg/μl of 17β-estradiol (E_2_) for a continuous infusion [50]. The pump flux was 0.25 μl/h and the infusion was continued for 3 weeks from the start of the experiment. In the third trial, they were tested 4 h after injection with 500 μg progesterone (Calbiochem) [9,50,51]. In each test, lordosis behavior of the female was recorded for 20 min for the mounting attempts of stimulus male. If no mounts occurred within 5 min, the female was placed with a different stimulus male.

### Test for masculine behaviour

In masculinization behavior tests, the female subject was placed alone in her own cage to adapt for 15 min. Subsequently, an E_2_-primed C57BL/6 partner female was introduced, and the number of mounts and intromission-like behaviors shown by the female subject were recorded for 30 min. The occurrences of behaviors were counted once the subject animal was separated by more than half the body length from its partner. The behaviors were scored by an observer uninformed of the experimental treatments.

### Testing urine preference

Eight to ten week-old female mice were injected daily for 3 days with 20 μg of 17β-estradiol [50]. The urines from E_2_-primed female and male mice (n = 8) were collected by holding the scruff of mouse during the 30 days of period in order to get sufficient volume, which were pooled and immediately stored in aliquots at -80°C until use [52]. The urine preference tests were normally carried out between 15:00 and 17:00. The tests were conducted in a clear acrylic container (700 × 200 × 200 mm), which was cleaned before testing, lined with a mix of clean and home-cage beddings, and all of the subjects were habituated in a clear acrylic partition (230 × 180 × 180 mm) placed in the center of the container for 20 min [53]. Olfactory stimuli soaked on 3 M paper disks (20 × 20 mm) with 100 μl of male or female urine were placed at both ends of the container. The test was initiated by removing the partition, and the times spent actively sniffing the urine samples were recorded during a period of 5 min.

### Immunocytochemistry for tyrosine hydroxylase

To determine tyrosine hydroxylase expression, each mouse was anesthetized with an injection of Avertin Anesthesia and killed by decapitation; their brains were quickly removed and fixed in 4% paraformaldehyde in 0.1 M phosphate buffer at pH 7.4. After dehydration, paraffin embedded sections (4 Ām) were rehydrated and microwaved in 10 mM citric acid buffer for 15 min, and washed three times in TBS. Endogenous peroxidase was blocked with 3% H_2_O_2 _for 10 min at room temperature. The sections were washed three more times in TBS and incubated for 1 h at room temperature in TBS containing 0.1% Tween 20 (TBST) with 3% bovine serum albumin. Each slide was incubated overnight at 4°C with rabbit polyclonal antibodies to tyrosine hydroxylase (Abcam, Cambridge, UK). Antibodies were diluted to working concentrations (1:750 or 1:500) in TBST containing 3% BSA. After 16 h at 4°C, the slides were washed three times for 5 min each in TBST before incubation with horseradish peroxidase-labeled goat anti-rabbit antibodies (Zymed, South San Francisco, CA) for 1 h at room temperature. The sections were washed three times for 5 min in TBST before application of an EnVision^® ^Dual Link HRP System (DakoCytomation, Carpinteria, CA). The development of a brown-colored reaction was stopped by rinsing them in TBST. To quantify the number of TH-positive neurons, all slides and images were coded so that the investigator had no knowledge on genotypes of the subjects, from which the immune-reactive cells were counted using Olympus BX60 microscope.

### 2D-electrophoresis and binding studies

The fetal mouse sera containing AFP were obtained by heart puncture of 25 each of wild-type and mutant embryos at E16.5. The female mice were caged overnight individually with males, and mating was verified by the presence of a vaginal plug appeared in the following morning (regarded as E0.5). Using 2D electrophoresis and Matrix-assisted laser desorption/ionization - time-of-flight mass spectrometer (MALDI-TOF-MS) analysis, we detected two discriminantal isoforms of AFP. 2D gel electrophoresis was performed using linear immobilized pH gradient strips (GE Healthcare, Uppsala, Sweden) with a pI range from 4-7. Following SDS-PAGE using 10% gels, proteins were either visualized using Coomassie blue staining or electrotransferred onto nitrocellulose membranes before probing with anti-AFP antibody (goat polyclonal, Santa Cruz Biotechnology). Differences in fucosylation of AFP were assayed by crossed affinity immunoelectrophoresis with biotin-conjugated *Aleuria aurantia *lectin (Vector Laboratories, Burlingame, CA) or biotin-conjugated *Ulex europaeus *agglutinin I (Vector Laboratories). Detection was performed using an ECL system (Amersham Biosciences) according to the manufacturer's instructions. Spot intensities were analyzed using the NIH Image J program.

The serum was incubated with ^3^[H]-estradiol (PerkinElmer Life and Analytical Sciences, Boston, MA) in a concentration of 10^-9 ^M to 2 × 10^-6 ^M in 20 mM Hepes pH 7.4, 50 mM NaCl and 20% glycerol (HNG buffer) for 1 h at room temperature [18,54]. Nonspecifically bound counts were subtracted from the total counts to yield specifically bound counts. A bound steroid was separated from a free one using dextran-coated charcoal (DCC, Sigma). Measurements were done in duplicate, and each experiment was repeated three times. The association constant (K_a_) and maximal binding capacity (B_max_) were estimated by the Scatchard plot analysis.

### Gene expression analysis

Total RNAs were isolated from mouse embryos by homogenizing with the TRI reagent (the manufacturer's protocol, Molecular research center). Prior to RT-PCR, aliquots of 4 μg of total RNA extracted from complete embryos were used as template for reverse transcriptase with oligo-dT Primers. Reverse transcription was carried out with RNase H^- ^Superscript II reverse transcriptase (Invitrogen). Real Time PCR reactions (20 μl) contained 10 μl of iQ SYBR Green Supermix (Biorad), 1 μl cDNA and primers (PrimerBank ID, 31982513a1; forward, 5'-CTTCCCTCATCCTCCTGCTAC-3'; reverse, 5'-ACAAACTGGGTAAAGGTGATGG-3'). For quantitative PCR, SYBR fluorescence was used to generate Ct value that was normalized to obtain absolute values using GAPDH as an internal standard. All assays were conducted in triplicates.

### Statistical analysis

Statistical comparisons were done using one-way analysis of variance (ANOVA) or repeated measures ANOVA, followed by Fisher's least protected significant difference test.

## Authors' contributions

DP and CP designed research; DP, DC and JL performed research; DP, DL, and CP analyzed data; and DP and CP wrote the paper. All authors read and approved the final manuscript
